# Review of Biosimilar Trials and Data on Etanercept in Rheumatoid Arthritis

**DOI:** 10.1007/s11926-018-0799-0

**Published:** 2018-11-09

**Authors:** Laura Chadwick, Sizheng Zhao, Eduardo Mysler, Robert J. Moots

**Affiliations:** 1 Institute of Ageing and Chronic Disease, University of Liverpool, Aintree University Hospital, Longmoor Lane, Liverpool, L9 7AL UK; 2 Organización Medica de Investigación, Buenos Aires, Argentina; 3 Department of Musculoskeletal Biology I, Institute of Ageing and Chronic Disease, Aintree University Hospital, Clinical Sciences Centre, Longmoor Lane, Liverpool, L9 7AL UK

**Keywords:** Etanercept, Biosimilar, Rheumatoid arthritis, ENBREL, Benepali, Erelzi

## Abstract

**Purpose of Review:**

Etanercept was the first tumour necrosis factor inhibitor approved to treat rheumatoid arthritis (RA) in the United States (US) and Europe. The recent patent expiration of the etanercept originator ENBREL in Europe has facilitated the development of biosimilar products, creating the prospect of reduced treatment costs. In this article, we review the original trials for etanercept in RA to facilitate critical appraisal of biosimilar trial data.

**Recent Findings:**

Two etanercept biosimilars are currently approved in Europe and/or the US, SB4 (Benepali) and GP2015 (Erelzi), having met the pre-specified equivalence criteria for biosimilarity. Trial data demonstrates subtle differences in clinical outcomes and adverse events between the biosimilars and the reference product (RP).

**Summary:**

The development of etanercept biosimilars may reduce the financial burden of treating RA, but real-world data regarding efficacy and safety in comparison to the RP will be vital to assess for meaningful differences.

## Introduction

Rheumatoid arthritis (RA) is a chronic autoimmune disease characterised by systemic inflammation which can lead to progressive joint damage and deformity if left untreated. Significant advances in RA treatment came with the introduction of biologic disease-modifying drugs (bDMARDs) such as tumour necrosis factor inhibitors (TNFi). The etanercept bio-originator, ENBREL, was the first TNFi to gain approval from the United States (US) Food & Drug Administration (FDA) and the European Medicines Agency (EMA) for the treatment of moderate to severe RA in 1998 and remains a first-line bDMARD therapy for RA worldwide. Additional licenced indications for etanercept include plaque psoriasis, psoriatic arthritis, ankylosing spondylitis, axial spondyloarthritis and polyarticular juvenile idiopathic arthritis [1].

Whilst the introduction of TNFi revolutionised RA treatment, the associated financial burden is significant; the cost per patient per year for ENBREL is £9295 in the United Kingdom (UK) [2] and estimated at $15,345 in the US [3]. There has been great interest in the development of molecules with biological similarity to bio-originator bDMARDs, known as biosimilars, as patent expiration for bio-originator TNFi’s began with ENBREL in 2015 in Europe (the patent in the US has been extended until 2028) [4]. The introduction of biosimilars into clinical practice will significantly improve the financial cost of RA treatment worldwide.

Biosimilar products must prove high similarity in safety, purity and potency to obtain regulatory approval [5, 6]. This can be achieved through phase I and phase III clinical trials in which the bio-originator and biosimilar are directly compared to confirm equivocal efficacy and safety against pre-specified margins [7]. However, subtle differences in efficacy and safety outcomes have been noted in key comparator trials, and the potential clinical implications for this in daily practice are yet to be established. Moreover, when a biosimilar product has obtained licencing for one indication for which the bio-originator is already approved, the licencing can then be extrapolated to all other licenced indications for the bio-originator without further head-to-head comparative clinical trials. On-going vigilance by clinicians in reporting adverse events (AEs) and treatment outcomes is therefore vital.

This article summarises the pharmacology and clinical efficacy of etanercept (ENBREL) to facilitate a critical review of the data regarding the safety and efficacy of etanercept biosimilars for RA, focusing on products approved by the EMA and FDA.

## Pharmacology of Etanercept

**Fig. 1:**
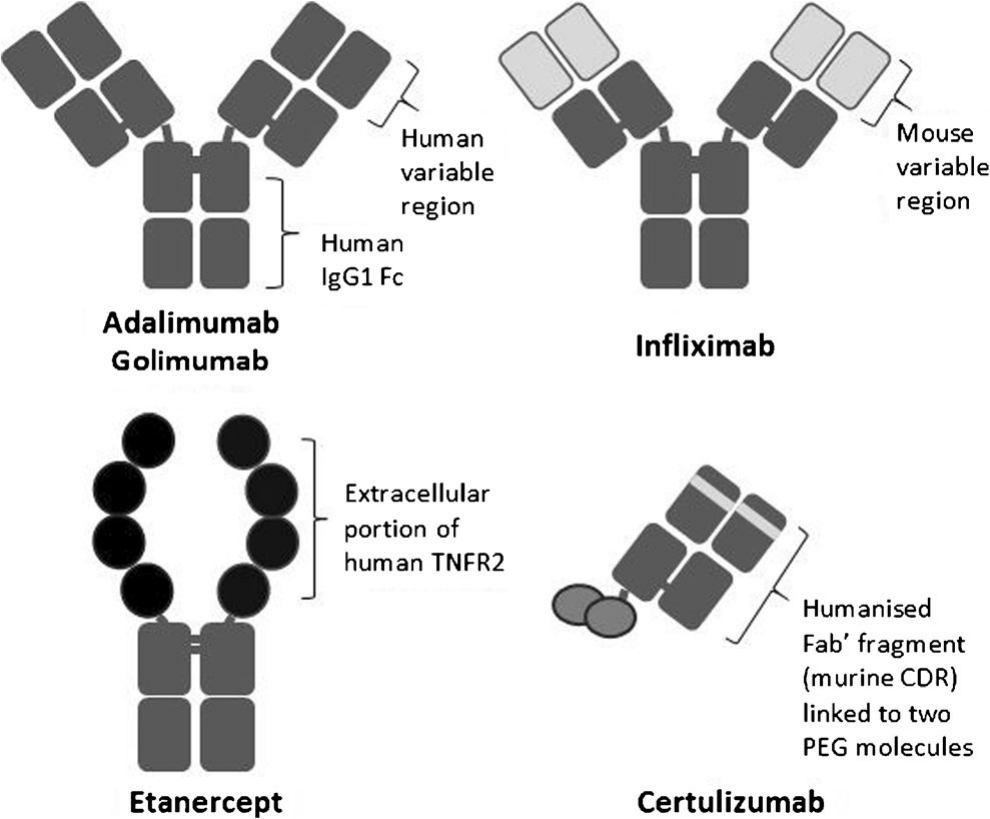
Etanercept structure compared with other TNFis. TNFR2 TNF receptor 2, Fc fragment crystallisable region, Fab′ antigen binding fragment, CDR complementarity-determining region, PEG polyethylene glycol

Etanercept is a fully human dimeric fusion protein consisting of the human Fc portion of IgG1 linked to the extracellular ligand-binding domain of the TNF p75 receptor, produced using recombinant DNA technology in a Chinese hamster ovary line cell line [8]. The structure of etanercept is depicted in Fig. 1.

Etanercept is administered via subcutaneous injection reaching peak serum concentration at 48–60 h with an elimination half-life of 70–100 h. The volume of distribution is small although it is able to penetrate the synovium [9, 10]. The mechanism of action is via binding to TNF, competitively inhibiting TNF from binding to cell receptors and preventing pathway activation [11]. The dimeric structure allows the molecule to bind to two TNF molecules at an affinity more than 50 times that of naturally occurring monomeric forms [8]. The immunogenicity of etanercept is reported to be very low, supported by a recent multi-national cross-sectional study in which no patients on etanercept (*n* = 200) developed anti-drug antibodies (ADAs) compared to 31% and 17% of patients on adalimumab and infliximab, respectively [12].

From this point onwards, “etanercept” in clinical trials refers to the reference product (RP) ENBREL unless otherwise stated.

## Clinical Efficacy of Etanercept

The clinical efficacy of etanercept for RA has been assessed in several randomised controlled trials (RCTs) and open label extensions [13–19] (Table 1). Clinical response was measured using a variety of outcome measures, commonly including American College of Rheumatology (ACR) response criteria (ACR20, 50 and 70 requires a 20%, 50% and 70% improvement in the defined response parameters respectively [20]), 28 joint count disease assessment score (DAS28) remission (< 2.6) and patient-reported physical disability assessed via Health Assessment Questionnaires (HAQ). Radiographic progression was measured by modified or original total Sharp score. Unless otherwise specified, the clinical trials below used a dose of etanercept 25 mg twice weekly, as non-inferiority to the current recommended dose of etanercept 50 mg weekly was not demonstrated until 2004 [16].

**Table 1:** Randomised controlled trials of etanercept in rheumatoid arthritis

Study	No. of participants	Disease duration	Treatment groups	Study duration	Outcomes assessed
Moreland 1999 [13]	246	Mean 12 years	ETN 10 mg twice/week ETN 25 mg twice/week Placebo twice/week	6 months	Primary endpoint: ACR20, ACR50 ACR 70, tender/swollen joint counts, pain VAS, physician/patient global, duration morning stiffness, HAQ Adverse events
Weinblatt 1999 [14]	89	Mean 13 years	ETN 25 mg twice/week + MTX Placebo twice/week + MTX	6 months	Primary endpoint: ACR20 ACR50, ACR70 Tender/swollen joint counts, pain VAS, physician/patient global, duration morning stiffness, HAQ Adverse events
Bathon 2000 [15]	632	< 3 years	ETN 10 mg twice/week + placebo ETN 25 mg twice/week + placebo Placebo twice/week + MTX	1 years	Primary endpoint: ACR-N, change in modified Sharp score ACR20, ACR50, ACR70 Adverse events
Keystone 2004 [16]	420	Mean 8.9 years	ETN 50 mg once/week + placebo ETN 25 mg twice/week Placebo twice/week (then ETN 25 mg twice/week after 8 weeks)	16 weeks	Primary endpoint: ACR20 ACR50, ACR70 Serum drug levels
Klareskog 2004 (TEMPO) [17]	686	6 months to 20 years	ETN 25 mg twice/week + MTX ETN 25 mg twice/week + placebo Placebo twice/week + MTX	3 years	Primary endpoint: ACR-N AUC; modified total Sharp score ACR20, ACR50, ACR70, DAS, DAS remission, HAQ Adverse events
Combe 2006 [19]	260	Mean 6.6 years	ETN 25 mg twice/week + placebo Placebo twice/week + SSZ ETN 25 mg twice/week + SSZ	2 years	Primary endpoint: ACR20 ACR50, ACR70, DAS44-ESR, morning stiffness duration HAQ, EQ-5D VAS Adverse events
Emery 2008 (COMET) [18]	542	3 months to 2 years	ETN 50 mg once/week + MTX Placebo once/week + MTX	2 years	Primary endpoints: DAS28 remission (< 2.6), change in van der Heijde modified total Sharp score HAQ, employment status Adverse events

*ETN* etanercept, *SSZ* sulfasalazine, *MTX* methotrexate, *AUC* area under the curve, *ACR-N* American College of Rheumatology N index of improvement, *VAS* visual analogue scale, *HAQ* Health Assessment Questionnaire

Etanercept, as monotherapy or in combination with MTX, has consistently been shown to be superior to placebo [13, 14, 16]. Etanercept monotherapy has shown relatively similar efficacy to MTX monotherapy in both patients with early RA (< 3 years duration; the TEMPO study) [15] and in patients with long-standing RA [17] with similar ACR20 responses at 52 weeks. However, in the TEMPO study etanercept monotherapy showed greater ACR responses at 24 weeks compared to MTX, suggesting a more rapid onset of improvement. In the TEMPO extension radiographic progression was lower in the etanercept group at 100 weeks with no significant infections or malignancies [21].

Etanercept-MTX combination therapy has consistently been shown to be superior to either etanercept or MTX monotherapy regarding clinical outcomes and radiographic progression with no significant difference in rates of serious infection or malignancy [14, 17, 18, 21, 22]. This includes the COMET study, with a patient population of early (< 2 years) moderate to severe RA who were MTX naïve and was the first RCT using the 50 mg weekly dose [18], the TEMPO study outlined above [17, 21] and patients with long-standing RA [14]. Similar findings with respect to monotherapy were also reported with regard to sulfasalazine (SSZ), although patients receiving etanercept experienced significantly higher rates of infections and non-infectious AEs compared to SSZ alone [19].

There are no head-to-head RCTs between etanercept and other bDMARDs. Multiple systematic reviews and meta-analyses comparing efficacy and safety of etanercept and other bDMARDs have been published [23–26]. A Cochrane review of previous bDMARD meta-analyses found similar efficacy between etanercept and adalimumab, infliximab, abatacept and rituximab, with etanercept seeming to cause fewer treatment withdrawals due to AEs than adalimumab or infliximab [27]. However, more recent Cochrane meta-analyses with the additional comparators of golimumab, certoliuzmab and tofacitinib reported no clinically meaningful differences in outcomes or withdrawal rates across four RA populations defined by prior treatment exposure and/or response [28–31]; safety profiles were largely comparable but insufficient to provide conclusions regarding risk of malignancy. Another recent systematic review found similar rates of serious AEs (SAEs) between etanercept and abatacept, adalimumab, golimumab, rituximab and tofacitinib with potentially higher rates of SAEs in certolizumab and tocilizumab in the first 6 months of treatment [32]. Regarding cost-effectiveness, a systematic review comparing etanercept, adalimumab and infliximab for the treatment of RA was favourable towards etanercept; the incremental cost-effectiveness ratio (ICER) for etanercept was £24,000 per quality of life year (QALY) in comparison to £30,000 per QALY for adalimumab and £38,000 per QALY for infliximab [33].

## Etanercept Biosimilars

Presently, the etanercept biosimilar SB4 (Benepali) is approved for use in RA by the EMA and GP2015 (Erelzi) is approved for use in RA by both the EMA and the FDA. Both SB4 and GP2015 phase III RCTs will be reviewed regarding clinical outcomes, safety profiles and trial characteristics with reference to the RCTs for the RP.

The etanercept biosimilar YLB113 has completed phase III trials and was submitted for EMA approval in May 2018 [34], but the data is not currently accessible via PubMed for this review. Phase III trial data are available for HD203 (Davictrel©) which was licenced in South Korea but subsequently withdrawn, LBEC0101 (Eucept©) licenced in Japan and South Korea, and CHS-0214 (in abstract form [35]) which is currently only marketed in the Caribbean and Latin America. Additional biosimilars that are not approved by the EMA or FDA but are available elsewhere worldwide includes Etacept (Cipla©) in India.

## SB4 (Benepali)

Samsung’s SB4 was approved by the EMA in June 2017. The manufacturer demonstrated that SB4 and the RP were highly similar in structure, function and pharmacokinetics in healthy male subjects [36]. For RA, a phase III, double blind, randomised equivalence study was performed comparing SB4 to the RP with published results at both 24 [37] and 52 weeks [38]. The trial included 596 patients with moderate to severe RA despite a minimum of 6 months MTX treatment without prior bDMARD exposure. All patients were required to take concomitant MTX (10–25 mg/week) throughout the trial period. The primary endpoint was ACR20 with an equivalence margin of − 15% to 15% in line with EMA guidelines [39].

The proportion of patients meeting ACR20 in the per-protocol set (PPS) was 78.1% for SB4 and 80.3% for the RP at week 24, and 80.8% for SB4 and 81.5% for the RP at week 52, demonstrating equivalence (results from the full analysis set (FAS) were similar). There was no significant difference in secondary endpoints between the two groups, although trends marginally favoured SB4 at week 52 (ACR50 58.5% vs 53.2%; ACR70 37.5% vs 31.0%, for SB4 and RP, respectively); mean improvement in DAS28 and HAQ also favoured SB4, as did mean change in total Sharp score (mTSS) for radiographic progression [38].

There were no statistically significant differences in rates of AEs between groups other than for injection site reactions (ISRs): 3.7% in the SB4 group compared to 17.5% in the RP group at week 52 (*p* < 0.001). Further analysis published separately regarding ISRs in relation to ADA status found no conclusive evidence to explain this difference, although the authors suggest that this may be related to differences in formation composition and material differences (unlike the RP, the SB4 needle does not contain l-arginine or latex) [40]. SAEs were slightly more common in the SB4 group at 6% compared to 5.1% in the RP, although only one SAE was treatment associated in the SB4 group compared to 6 in the RP group. Although not included in the RCT manuscript, the EMA Assessment Report noted a difference between groups when AEs are grouped in to hepatobiliary disorders, but was concluded by the EMA not to be treatment related. Additionally, there were four malignancies in the SB4 group compared to one in the RP group, but the EMA remarked that these numbers were too low to conclude on significance [41]. Regarding immunogenicity, incidence of ADAs was significantly lower in the SB4 group at week 52 (1% compared to 13.2%). However, only one RP patient developed ADA titres of neutralising capacity with the majority of ADAs being transient; therefore, the clinical relevance of this is unclear, particularly over longer periods of treatment [38].

In the open label extension, a smaller subset of patients in the RP arm were switched to SB4 for a further 52 weeks. This demonstrated sustained and comparable clinical efficacy and safety outcomes between groups, suggesting that there are no adverse outcomes associated with switching from the RP to SB4. One patient per group developed non-neutralising levels of ADAs [42].

## GP2015 (Erelzi)

GP2015 from Sandoz was approved by the FDA in 2016 and the EMA in 2017. The pharmacokinetics of GP2015 were shown to be highly similar to the RP in structure, function and pharmacokinetics in healthy male subjects [43]. The approval for GP2015 in RA has been extrapolated from the EGALITY study, comparing GP2015 and the RP in 531 patients with plaque psoriasis which met the primary endpoint [44]. Patients were randomised at 12 weeks to continue treatment or to switch between products on a six weekly basis for a further 18 weeks without a negative impact on efficacy or an increase in ADAs.

Similarly to SB4, ISRs were higher in the RP group compared to GP2015 (14.2% vs 4.9%). There were no clinically meaningful differences in AEs or SAEs, although overall rates of treatment emergent AEs of special interest, including herpes infection, fungal infections, neutropenia and other dermatological diagnoses (detailed in the study’s supplementary material) were higher in the continued GP2015 group vs. continued RP group (11% vs. 4.7%) and in the switched GP2015 vs switched RP group (11% vs 5%). Regarding immunogenicity, no patients in the GP2015 developed ADAs. Four patients in the continued RP and one patient in the RP switchers group developed ADAs, all of which were transient [44].

The EQUIRA study comparing RP and GP2015 for moderate to severe RA with an inadequate response to MTX was completed in June 2017 [45] with week 24 results published in abstract form (ACR20 88.8% vs 93.6%, ACR50 63.9% vs 71.2% and ACR70 33.7% vs 42.9% for GP2015 and RP, respectively) [46]. The primary outcome of mean change from baseline of DAS28-CRP was within the pre-specified equivalence margin. The rates of SAEs were 0.5% vs 3.2% for GP2015 vs RP with similar findings as reported for SB4 regarding frequency of ISRs: 7.0% to 17.9% for GP2015 and RP, respectively. There were low levels of transient ADAs detected with no significant levels present in either group at week 24. The full manuscript is required to review this data in more detail. Recently, abstract data regarding switching between RP and GP2015 was published with no significant impact on efficacy or safety at week 48 [47].

## HD203

HD203 was approved for use in South Korea but upon request of the manufacturer was subsequently withdrawn from the market after the manufacturing facility was sold. Following publication of pharmacokinetic data demonstrating biosimilarity [48], a phase III multicentre double-blinded RCT was performed in South Korea comparing the RP at 25 mg twice weekly to HD203, both in combination with MTX. The proportion of patients achieving ACR50 was statistically significantly greater in the HD203 group at week 24 and week 48 in the PPS (and at week 24 in the FAS) but not ACR20 or ACR70 at any time point; therefore, this is unlikely to be of clinical significance. In the PPS dataset, the response rates were as follows for HD203 and the RP: ACR20 87.3% to 86.5%, ACR50 68.2% to 54.5%, and ACR70 38.2% to 33.9% (reported in the manuscripts supplementary data).

Overall, there were no significant differences in clinical outcomes, safety or immunogenicity between the groups. The difference in rates of ISRs between groups was less significant than for the two biosimilars outlined above; 2% compared to 5.5% for HD203 and the RP, respectively [49].

## LBEC0101

At the time of writing, LBEC0101 has been approved for use in Japan and South Korea but not by the EMA or FDA. Following publication of pharmacokinetic data demonstrating biosimilarity [50], a phase III multicentre double-blinded RCT was performed in Japan and South Korea comparing the RP at 50 mg weekly to LBEC0101, both in combination with MTX [51]. Patients who had been previously exposed to ≥ 2 bDMARDs or to etanercept were excluded; 16% of patients had previous bDMARD exposure. However, no exclusions were made based on comorbidity other than active tuberculosis. The primary endpoint of mean change in DAS28-ESR from baseline at week 52 was − 3.01 (95% CI − 3.198, − 2.820) in the LBEC0101 group and − 2.86 (95% CI − 3.051, − 2.667) in the RP group, meeting the pre-specified equivalence margin of − 0.6 to 6. ACR20, 50 and 70 responses comparing LBEC0101 to RP at week 52 were as follows: 92.0% vs 88.4%, 74.7% vs 65.8% and 58.0% vs 50.0%.

Rates of AEs are much higher in this study than reported elsewhere: 92% in the LBEC0101 group and 92.5% in the RP group. Rates of SAEs were 16.6% and 10.7% respectively and although rates of SAEs related to treatment were 7% in both groups. Of note, rates of hepatic function abnormalities were doubled in the LBEC0101 group at 6.4% compared to 3.2%, although the definition of abnormal hepatic function is not described. Again, ISRs were more frequent in the RP group at 34.2% compared to 10.2%, and ADAs were more frequent in the RP group at 9.6% compared to 1.6%.

## Equivalence and Switching

It has been noted that clinical responses in biosimilar and RP comparator trials may report different responses for the RP than reported in pivotal RP trials. For example, PASI75 response of the etanercept RP group (76%) in the EGALITY study was much higher than in previous RP studies (47% to 49%) [44, 52, 53].

**Fig. 2:**
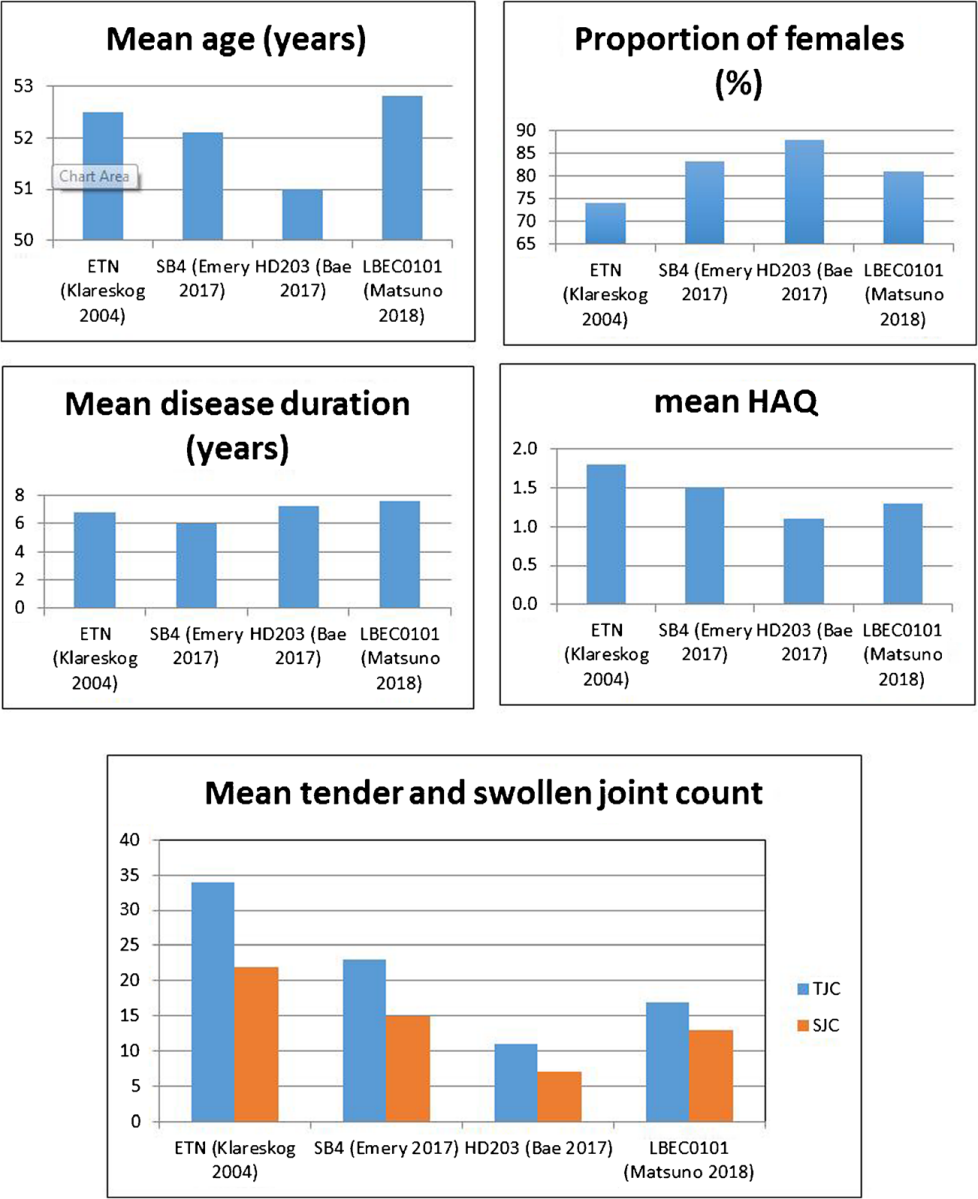
Differences in patient characteristics between pivotal RP and biosimilar etanercept trials (ETN, etanercept RP)

The pivotal RCTs for etanercept outlined above reported variable ACR20 responses across a variety of time points, with varying patient populations and previous/current treatment exposure, making comparisons difficult (Fig. 1). All etanercept biosimilar trials compared the biosimilar and RP in combination with MTX due to pivotal RP trial data supporting greater clinical responses in combination therapy. The RCTs for both SB4 and HD203 chose patients who have failed on ≥ 6 months of MTX. The two trials have reasonably comparable baseline data regarding age, gender and disease duration although the HD203 trial patients had a lower baseline swollen joint count (SJC), tender joint count (TJC) and HAQ than the SB4 trial patients. Interestingly, the performance of the RP is relatively similar in these two trials (Fig. 2). Comparing 48-week RP data in the HD203 trial to the 52-week RP data in the SB4 trial is as follows: ACR20 81.5% to 86.5%, ACR50 53.2% to 54.5% and 31.0% to 33.9% [38, 48].

**Fig. 3:**
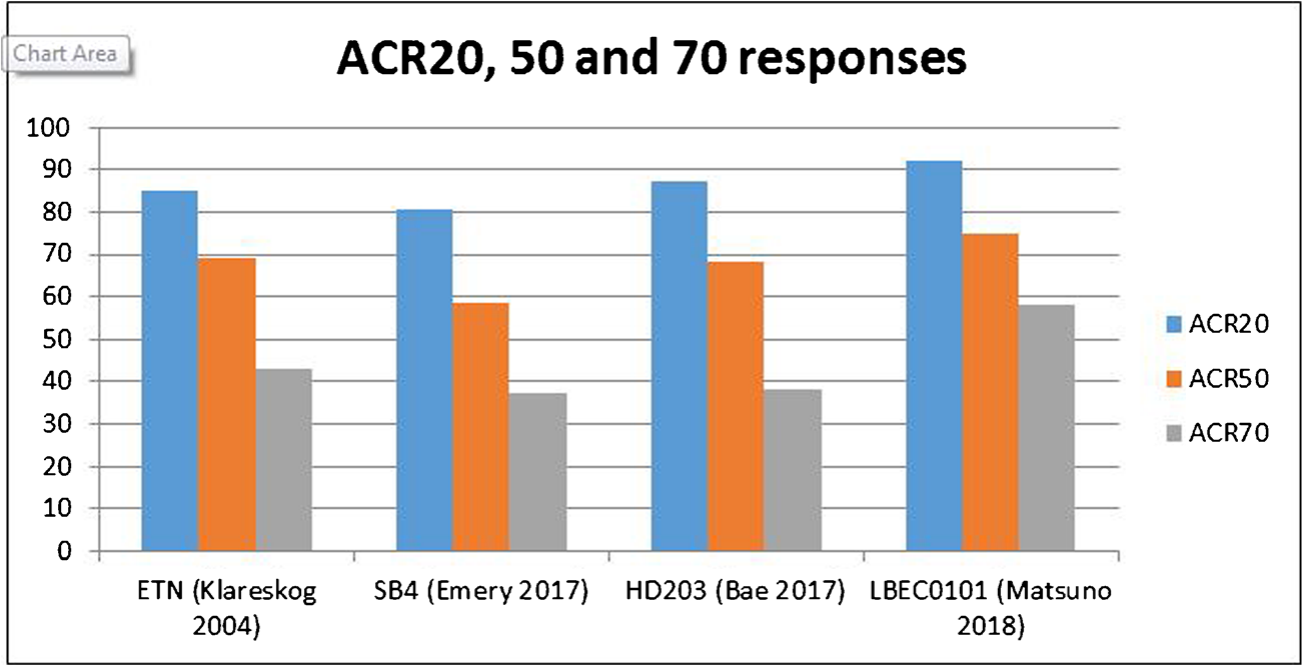
Differences in ACR response rates for the RP in pivotal and biosimilar etanercept trials (ACR70 not reported for ETN Moreland 1997)

Higher ACR responses were reported for the RP in the LBEC0101 RCT with rates of ACR20, 50 and 70 reported at 88.4%, 65.8% and 50%, respectively. This cannot be explained fully by ethnicity, as the HD203 RCT was also conducted in Asia, although the majority of recruitment sites in the LBEC0101 RCT were in Japan as opposed to South Korea. The higher ACR responses for RP are somewhat surprising given that 16% of patients in this trial had previous bDMARD exposure, as patients with prior bDMARD failure are less likely to respond to a subsequent bDMARD [54, 55], although the reason for previous bDMARD discontinuation (inefficacy or AE) was not discussed. The rates of AEs and SAEs were also notably higher in this RCT, which could be linked to the exclusion criteria not featuring comorbidities in contrast to the other RCTs discussed (Figs. 2 and 3).

The RP RCT most similar to the biosimilar RCTs is the TEMPO study, which assessed RP and MTX combination therapy, although patients with MTX exposure in the prior 6 months or previous MTX failure were ineligible and the patients had a significantly greater baseline SJC and TJC than in the biosimilar RCTs. The ACR20 response of 85% at 52 weeks was comparable to the RP data from biosimilar trials, whereas the ACR50 and ACR70 responses were significantly higher at 69% and 43%, respectively [17]. In all other pivotal RP trials measuring ACR20, responses for RP were significantly lower, but the majority were studying monotherapy. Only one trial reporting ACR20 used combination therapy, reporting ACR20, 50 and 70 at 71%, 39% and 15%, respectively, but featured significantly younger patients with much greater disease duration than other RP trials and featured only 89 patients, limiting the ability to make comparative comments [14].

With only one phase III RCT for each biosimilar, it is difficult to draw meaningful conclusions regarding the risk of SAEs, with severe infections and malignancy being rare events in both the biosimilar and RP groups. Additionally, these RCTs have stringent inclusion and exclusion criteria which are not reflective of real-world practice and limits the generalisability of these findings. Data regarding ISRs and development of ADAs does appear to favour biosimilar etanercept use over RP. This is additionally supported by an international survey of 149 nurses which reported preference for the SB4 autoinjector over the RP autoinjector for ease of use [56]. However, patient preferences regarding switching or remaining on their established treatment will differ and must be taken into account. Although not in a RA patient group, the switching between biosimilar and RP in the EGALITY trial did not appear to alter AE profiles or immunogenicity, supporting the safety of switching where appropriate, and clinical outcomes and safety/immunogenicity profiles were sustained in the SB4 switching open label extension study [42].

## Conclusion

Biosimilar etanercept products are now available to RA patients in numerous countries, with the potential to significantly reduce the financial burden of treating RA worldwide. Robust trial evidence has demonstrated that SB4 and HD203 meet pre-specified biosimilarity criteria and are non-inferior to the RP for RA, with similar results for GD2015 in plaque psoriasis. The ability to extrapolate licence indications without data supporting the use of that product in certain indications may create uncertainty for prescribers. Real-world efficacy and safety data via pharmacovigilance studies will therefore be essential to gather clinical evidence of the benefits and risks of switching in all patients, such as the BENEFIT trial monitoring patients switching between the RP and SB4 [57]. The continued development and approval of biosimilar products for etanercept and other bDMARDS offer exciting prospects regarding future access and opportunities in the treatment of RA worldwide.
